# Exploring the contribution of integrated healthcare practices to malaria control in Ghana: perspectives of medical herbalists

**DOI:** 10.1186/s12906-025-04746-9

**Published:** 2025-01-14

**Authors:** Irene G. Ampomah, Susan Devine, Genevieve A. Ampomah, Theophilus I. Emeto

**Affiliations:** 1 Department of Population and Health, University of Cape Coast, UC 182, Cape Coast, Ghana; 2 Public Health and Tropical Medicine, James Cook University, Townsville, QLD 4811 Australia; 3 Department of Sociology, Kwame Nkrumah University of Science and Technology, Kumasi, Ghana; 4 World Health Organization Collaborating Centre for Vector-Borne and Neglected Tropical Diseases, James Cook University, Townsville, QLD 4811 Australia

**Keywords:** Ghana, Integrated healthcare, Malaria, Providers, Traditional herbal medicine

## Abstract

**Background:**

The integration of herbal and orthodox medicines has gained momentum in global health, ensuring improved management of infectious diseases like malaria. This study explored the experiences of medical herbalists working in Ghana’s diverse ecological zones to understand the contributions of integrated healthcare to malaria control.

**Methods:**

A phenomenological design was employed to conduct in-depth interviews with 19 purposively sampled medical herbalists. Framework analytical approach and Donabedian’s conceptual framework for quality of care were utilised in analysing the data.

**Results:**

Findings revealed high awareness of integrated healthcare practices among participants. Medical herbalists perceived integrated care as instrumental in enhancing malaria management through factors such as improved quality assurance, increased accessibility to integrated health facilities, patient-centred care, follow-up practices, and opportunities for continuous professional development. However, structural and process-related challenges were identified, including inadequate healthcare personnel, medicines, and equipment. Additionally, limited promotional activities, non-comprehensive National Health Insurance Scheme (NHIS), and ineffective referral systems were recognised as barriers hindering the effectiveness of the integrated system and its potential contribution to malaria control.

**Conclusion:**

Although national and health system-based challenges have thwarted the importance of integration on malaria control, medical herbalists feel optimistic about the intervention. To optimise the effectiveness of integrated healthcare in controlling malaria in Ghana would require policy modification and implementation. Future research could focus on developing healthcare frameworks, particularly for malaria, that prioritise quality service delivery within an integrated system.

**Supplementary Information:**

The online version contains supplementary material available at 10.1186/s12906-025-04746-9.

## Introduction

Malaria remains a major public health concern, particularly in Sub-Saharan Africa, where it accounts for millions of deaths annually [1]. Approximately 70% of the global malaria burden is concentrated in 11 African countries, including Ghana [2]. Despite Ghana’s progress in reducing severe malaria cases and achieving a 13% decline in childhood mortality [2], the disease still exerts a substantial strain on orthodox healthcare facilities. Considerable international investment from the Global Fund, World Bank, and bilateral donors has targeted malaria control, primarily promoting the use of orthodox anti-malarial drugs [1, 3, 4]. However, the high cost of these medications has hindered effective malaria control efforts in Ghana [5]. In contrast, the Ghanaian population has a documented history of utilising traditional herbal medicine (THM) for malaria treatment [6, 7]. Factors influencing this choice include dissatisfaction, perceived ineffectiveness, or adverse effects associated with orthodox treatments [8], as well as alignment with cultural beliefs and health philosophies [9]. Thus, THM plays a vital role in the Ghanaian healthcare system, particularly for malaria.

Recognising the significance of THM, the Ghanaian government has integrated it into the formal healthcare system through policy formulation, the establishment of THM clinics within select public hospitals, and the creation of a THM Department at Kwame Nkrumah University of Science and Technology (KNUST) [10, 11]. This allows individuals to access certified THM services and products (herbal products manufactured by Centre for Scientific Research into Plant Medicine [CSRPM] and approved by the Food and Drug Authority [FDA]) within public health facilities (integrated hospitals and private THM clinics) for malaria treatment [11]. Integrated hospitals as used in this study refer to government-owned hospitals with THM clinics while private THM clinics are owned and operated by individuals.

Despite this integration, no research to date has assessed its contribution to malaria control. This study aims to explore the perceived effects of integrated healthcare practices on malaria control in Ghana, specifically through the experiences of medical herbalists in the country’s coastal, forest, and savannah regions. We aim to:

1. Investigate the positive contributions of THM integration to healthcare service delivery for malaria control as perceived by medical herbalists.
2. Examine the challenges associated with the practice of integration and its impact on malaria control.

### Theoretical framework

**Fig. 1:**
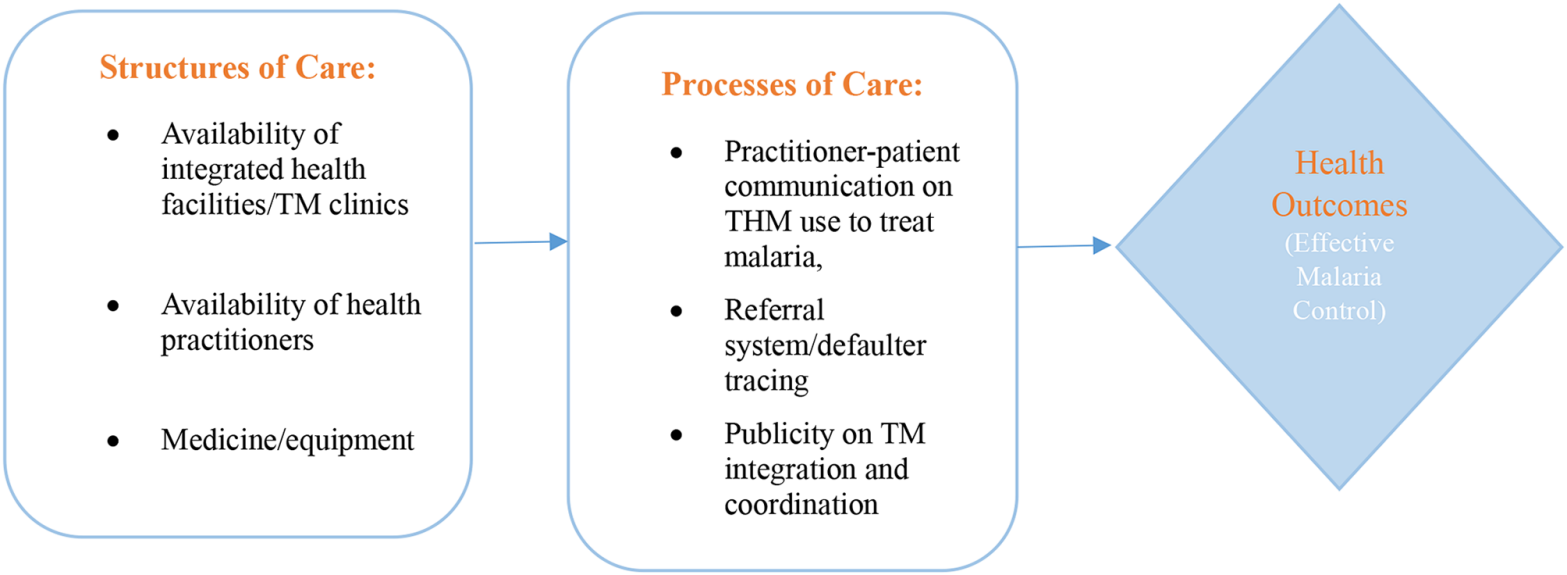
Donabedian’s framework for evaluating quality of healthcare. **Source**: Adopted from Donabedian [12]

Donabedian’s framework for evaluating healthcare quality guided this research [12]. This framework identifies three key elements - structure, process, and outcome (Fig. 1) [12], and emphasises their interrelationship in assessing healthcare quality [13]. Structure refers to the physical settings in which health services are delivered, including factors such as facility availability, healthcare personnel, and equipment. Process encompasses the delivery of healthcare services, such as referrals and patient follow-up. Finally, outcome reflects the impact of healthcare on patient populations, with positive outcomes reflecting reduced malaria prevalence/cases [12, 14]. While limited research has employed Donabedian’s framework in the context of malaria control interventions, its application here is intended to identify and understand how structural and process factors influence malaria control outcomes (positive or negative) within the integrated THM and orthodox medicine system in Ghana.

## Methods

### Study setting

**Fig. 2:**
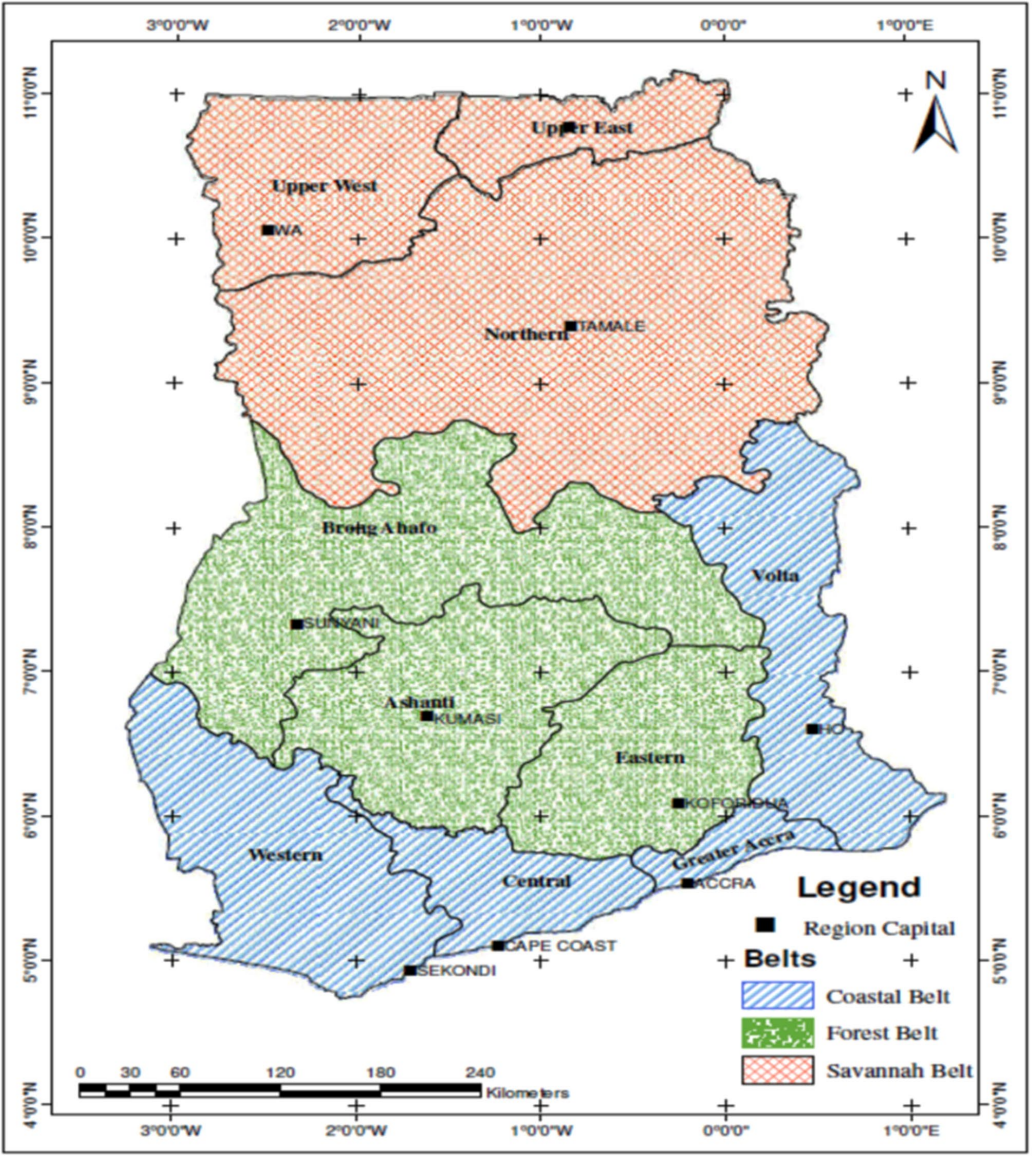
Map of Ghana showing specific study sites. **Source**: Bawa, Brobbey [15]

Ghana’s diverse ecological landscape can be broadly categorised into coastal, forest, and savannah belts (Fig. 2) [15]. To represent these variations, the Central (coastal), Ashanti (forest), and Upper West (savannah) regions were selected. These regions experience high malaria prevalence (15–20% in Ashanti, 20–25% in Upper West, and 25–30% in Central) [16].

The presence of integrated healthcare facilities and private THM clinics in these regions makes them suitable for exploring the effects of integration on malaria management. Within each region, the respective capitals (Kumasi, Cape Coast, and Wa) were chosen as specific study sites (Fig. 2). These capitals are endowed with public hospitals with THM units as well as privately owned THM clinics [17].

### Design

This qualitative study employed a phenomenological design to delve into the lived experiences of medical herbalists regarding integrated healthcare practices and malaria control. The design is well-suited for understanding lived experiences and allowed us to explore the “what” and “how” of the participants’ experiences with integration’s contribution to malaria control, ultimately providing a collective understanding of their encounters [18].

We extracted this paper from a larger study that explored the contribution of integrated healthcare to malaria control by gathering the experiences of health practitioners (medical doctors, pharmacists and medical herbalists) in the coastal, forest and savannah regions of Ghana. Other aspects of the research have been submitted for publication elsewhere.

### Study population

The study population consisted of experienced medical herbalists aged 18 or older who were practicing in integrated health facilities or private THM clinics in the Kumasi, Cape Coast metropolis and Wa municipality. Participants were required to be at least 18 years of age to ensure their capacity for informed consent, consistent with Ghanaian legal and ethical standards [19]. Medical herbalists were chosen as key stakeholders in the Ghanaian healthcare system, playing a crucial role in providing alternative healthcare services for malaria.

### Sample size and sampling technique

Purposive sampling was employed to recruit participants from 16 healthcare facilities within the designated regions. These facilities included six integrated hospitals and ten private THM clinics. Participant selection was primarily guided by specific criteria, such as the age, type of facility (integrated hospital or private THM clinic) and geographic location. This approach aimed to enhance the credibility of findings by ensuring variation in the sample.

The recruitment process utilised strategies such as direct contact and leveraging existing networks. All medical herbalists approached, agreed to participate in the study, providing detailed information that facilitated a comprehensive understanding of the research topics. Data saturation guided the sample size, with recruitment ending at 19 participants when no further new information or themes were observed in the data.

### Data collection period

Data collection spanned three months (July - October 2023). Three research assistants (two males and one female), holding master’s degrees in public health and with expertise in qualitative research, were recruited from the University of Cape Coast (UCC) and University of Development Studies (UDS) to assist with data collection. A training session (both online and in-person at UCC campus) was conducted in the fourth week of June 2023 to ensure they understood the study objectives and interview guide. Each session lasted four hours.

### Data collection procedure

Before actual data collection, three pilot interviews were conducted by the assistants and reviewed by IGA to ensure clarity of the questions and precision of data. Face-to-face, in-depth interviews served as the primary data collection method, utilising a semi-structured interview guide developed based on Donabedian’s framework for healthcare quality evaluation (See Supplementary file 1, Study Instrument). English was the primary language used, and interviews were conducted in participant-preferred, comfortable environments. All interviews were audio-recorded and lasted 45–60 min.

Following participant identification, information sheets explaining the study’s objectives, benefits, and ethical considerations were provided. Informed consent was obtained from each participant before the interview commenced. To mitigate bias and ensure interviewers adhered to the interview guide, a training manual was developed to guide question phrasing. The first author (IGA) observed the first three interviews to ensure consistency in the interview procedure.

To uphold anonymity, participants were assigned codes instead of identifiable information. Basic demographics (sex, age, specialty, and facility type) were collected at the beginning of interviews. The interview guide covered topics centred on participants’ perceptions and experiences regarding malaria management through THM integration in Ghana. Specifically, discussions focused on the perceived contributions of integration to malaria control and challenges associated with it. Data saturation was achieved when no new information or themes emerged after the 16th interview. However, to avoid inadvertent exclusion of new perspectives, the assistants interviewed three further interested participants who had previously expressed interest. Additionally, the assistants documented their observations during data collection. While repeat interviews were not necessary, interpretations were sought from some participants after the interviews had been transcribed.

### Data analysis

An experienced transcriber transcribed the audio-recorded interviews, with IGA reviewing them for accuracy. Transcribed data were then shared with some participants during follow-up meetings for verification and potential modifications. A framework analytical approach, involving both inductive and deductive techniques, was employed to analyse the transcribed data in NVivo software (version 12) [20].

Two authors (IGA and GAA) independently conducted data analysis. The transcribed data were thoroughly read by both researchers to become familiar with the content. Following this, a thematic framework outlining core themes and concepts was developed based on notes taken during familiarisation. This stage primarily employed inductive analysis, allowing themes to emerge directly from the data. Indexing involved systematically marking relevant data segments under identified themes. Here, an inductive approach was again utilised to ensure themes remained grounded in the data.

Next, charting involved organising the coded data into charts according to the identified themes. Finally, the mapping and interpretation stage involved arranging the charted data to illustrate the participants’ experiences with THM integration and its impact on malaria management in Ghana. Deductive analysis was utilised during mapping and interpretation, grouping themes under the components of Donabedian’s framework for healthcare quality evaluation.

To further ensure rigorous data analysis, introductory coding and theme generation were initially performed independently by IGA and GAA. Discrepancies in coding were resolved through discussion and consensus during a dedicated meeting. Additionally, one author (TIE) reviewed the finalised themes and quotes to enhance the trustworthiness of the research findings.

The trustworthiness of the research was further bolstered through various strategies, including:

- Supervisor/peer debriefing: Regular discussions with the supervisor (TIE) and peer researchers ensured a balanced perspective and identification of potential biases.
- Member checking: Sharing transcribed interviews with some participants for verification and potential modifications allowed for participant validation of the interpretations.
- Researcher/assistant triangulation: Consistency in data collection and interpretation was maintained through discussions between the lead author (IGA) and research assistants.
- Thorough description of study setting and methods: Extensive detail regarding the study context and methodological approach provides transparency and facilitates replicability [21, 22].

The identified themes are presented in the results section, accompanied by illustrative quotes and participant location/setting (e.g., Participant 1, Cape Coast). The Consolidated Criteria for Reporting Qualitative Studies (COREQ) checklist [23] was used to appraise the final version of the manuscript (See Supplementary file 2, COREQ Checklist).

## Results

Nineteen medical herbalists participated in the study, with ages ranging from 24 to 57 years (mean = 35 years). The majority (*n* = 16, 84.2%) were males. The sex imbalance is consistent with the lower number of female medical herbalists practicing in Ghana compared to males. Nearly half (*n* = 9, 47.4%) practiced in integrated healthcare facilities, while the remainder operated in private THM clinics. All participants were general practitioners, treating various ailments including malaria. The coastal belt (Cape Coast) had the highest representation (*n* = 9, 47.4%) among participants (Table 1).

**Table 1:** Characteristics of study participants (*N* = 19)

Participants’ characteristics	Frequency (*n*)	Percentage (%)
**Sex**		
Male	16	84.2
Female	3	15.8
**Participants’ affiliated facilities**		
Integrated hospital	9	47.4
Private THM	10	52.6
**Place of operation**		
Savannah	5	26.3
Forest	5	26.3
Coastal	9	47.4

### Themes

Thematic analysis of participant narratives revealed five key themes (Table 2):

1. Benefits of THM Practice: This theme explored the perceived advantages of THM use.
2. Knowledge/Understanding of THM Integration: This theme examined participants’ understanding of the integrated healthcare model.
3. Structure/Infrastructure for Integrated Care Delivery: This theme addressed the physical resources and settings associated with integrated healthcare delivery.
4. Processes/Activities in Integrated Healthcare Delivery: This theme described the activities and workflows involved in the integrated care model.
5. Outcomes/Consequences of THM Integration on Malaria Control: This theme investigated the perceived impact of THM integration on malaria management in Ghana.

**Table 2:** Thematic table

Main theme	Sub-theme
Benefits of THM practice	Efficacy of THM Minimal side effects Employment creation
Knowledge/understanding of the practice of THM integration	
Structure/infrastructure associated with integrated healthcare delivery	Availability of integrated health facilities Quality assurance Inadequate health personnel Insufficient equipment and medicine
Process/activities relating to the delivery of integrated health services	Health system-based challenges • Orthodox medicine providers’ disapproval of THM • Ineffective referral approach National/general challenges • Inadequate promotional activities on THM integration • Non-exhaustive NHIS Patient-centred care Follow-ups THM training and research
Outcome/consequence of the practice of THM integration on malaria control	Reduced pressure on orthodox medicine providers Evidence-based THM practice leading to proper management of malaria

### Benefits of THM practice

Participants highlighted various advantages of THM practice, including its perceived efficacy, minimal side effects, and job creation potential. Participants believed THM offers faster malaria cures due to the body’s familiarity with such remedies. Others acknowledged that although all medications have side effects, those associated with THM were considered negligible. Additionally, some participants viewed the traditional health system as a source of employment, with private THM clinics creating job opportunities for the populace. The quotes that follow expatiate on the views expressed by participants regarding the merits of THM practice in Ghana:

### Efficacy of THM

*I can say herbal medicine cures malaria better than orthodox medicine. Our human system knows herbal medicine. It is like introducing food to your baby…. Our system accepts herbal medicine well* [Participant 12, Cape Coast].

### Minimal side effects

*All medications have side effects. Once it is an external thing that you are introducing into your body, there will be some side effects. But it is up to the degree of the side effects. When you compare the side effects, it is less for herbal medicines than the orthodox medications* [Participant 11, Cape Coast].

### Employment creation

*…THM has created jobs in Ghana. For example, when you go to Kumasi, there are a lot of herbal clinics/outlets that have been established and these clinics have and keep employing people* [Participant 1, Cape Coast].

### Knowledge/understanding of the practice of integrated healthcare

Participants generally demonstrated awareness of THM integration into the Ghanaian healthcare system. They specifically mentioned the establishment of a Traditional Herbal Medicine program at KNUST as evidence of this integration.

*I am aware of the integration. Perhaps, that is why they have started teaching THM at KNUST. We have students from ‘Tech’ that are studying THM as a course to become professionals* [Participant 5, Cape Coast].

This suggests that participants perceive the inclusion of THM within the formal education system as a significant step towards its legitimisation and integration within mainstream healthcare delivery.

### Structure/infrastructure associated with integrated healthcare delivery

This theme addressed the physical resources and settings associated with integrated healthcare delivery. Four key sub-themes emerged:

#### Availability of integrated health facilities

Participants acknowledged the presence of THM units within some government hospitals. However, they expressed concern about the limited number of these facilities. They attributed this inadequacy to factors such as low public awareness, insufficient government support, and consequently, low patient utilisation of integrated healthcare services.

*…we have 55 piloted facilities. That number is small compared to the number of orthodox healthcare facilities in the country. That also accounts for the unpopularity of the THM unit in the country because the more there are herbal medicine facilities, the more people will get to know and patronise such facilities* [Participant 11, Cape Coast].

*I am aware of the availability of integrated facilities. We have Tafo government hospital, Juaben government hospital, Bekwai, and in Accra we have LEKMA hospital, Police hospital, Tema general hospital, and then the Tamale teaching hospital, Ho government hospital, and Wawra government hospital in the Oti region. So, it is all over the country* [Participant 14, Kumasi].

*In the Upper West region, there is one there. That is, the Wa municipal hospital. There are about two medical herbalists at the Wa Municipal hospital* [Participant 4, Wa].

### Quality assurance

Participants practicing within integrated health facilities highlighted their commitment to quality healthcare delivery. They described adhering to established medical principles and protocols for patient care which included prescribing only herbal products listed on the Ministry of Health’s recommended herbal medicine list. Additionally, some participants viewed regular inspections by the FDA as a means to ensure the quality, efficacy, and safety of the herbal products and services they provide.

*We have the recommended herbal medicine list. So, the recommendation should be based on the list from the Ministry of Health which has been certified so we stick to defined medical principles* [Participant 2, Wa].

*These days, once you are a certified THM provider, the FDA will come for inspection and check that your facility is following scientific methods to ensure quality, effectiveness, and safety to those who would use it* [Participant 6, Cape Coast].

### Inadequate health personnel

Participants highlighted a shortage of qualified healthcare personnel within integrated healthcare facilities. They described performing multiple roles beyond their core duties, including procurement, reporting, medication request follow-up, and drug dispensing. This role overload was attributed to the limited number of staff and a perceived lack of personnel with expertise in herbal medicine. Furthermore, some participants suggested that existing medical herbalists might be drawn to other career paths, such as academia or positions with the Ghana FDA, potentially exacerbating the staffing shortage.

*In our case, we must initiate, follow up with memo to request for medications. They say they don’t know much about herbal medicines, so I must take charge and lead the procurement. So, you become part of pharmacy because you have to monitor and make sure they are dispensing the right drugs. So you move up and down; it is a lot of work because one person you are involved in reporting, payment process, monitoring, and everything* [Participant 2, Wa].

*…comparing to the country’s population, I don’t think we have enough qualified medical herbalists. The whole of Cape Coast metropolis, we are just five. Can you imagine!* [Participant 8, Cape Coast].

*The numbers are not enough…. we started herbal medicine in KNUST in 2001 and most of us don’t practice. They enter a different sector like FDA. Those who really practice is very few. They just branch into a different field. Some are in academia and the FDA* [Participant 17, Kumasi].

### Insufficient medicines and equipment

This sub-theme emerged primarily among participants from the forest belt. They reported experiencing disruptions in service delivery due to a shortage of essential herbal medicines. They attributed this shortage to cumbersome procurement processes perceived as overly bureaucratic. Additionally, delays in deliveries from approved manufacturing centres were identified as contributing to stockouts. These shortages reportedly hampered their ability to provide quality care.

In contrast, participants from the coastal belt highlighted the issue of inadequate equipment, particularly a lack of computers. This lack of technology was perceived as hindering their productivity and overall job satisfaction. They associated this equipment deficiency with limited or absent funding for integrated healthcare facilities.

*We sometimes run out of drugs because to order the drugs must pass through so many bureaucratic lines. When I talk about the bureaucratic system, I mean that it must go through many offices before it gets approved. Over here, they will bring the pro forma invoice then we work on it and get the voucher from the account. Then the signatory must come from different people. So, if one signatory has travelled, it becomes a challenge to order the drugs, hence limited or no drugs* [Participant 16, Kumasi].

*When we order our medicines from Akuapem Mampong, it takes a long time. It delays and that doesn’t help because the client needs it, which disturbs you too. Why should you delay in giving treatment to someone suffering from malaria?* [Participant 15, Kumasi].

*… We don’t have computers to assist with our work. And today, everything is e-health and so, if I do not come with my personal computer, then it means that I will be unable to work. Computer is a basic equipment that we think if we had, it would improve our healthcare delivery, but we don’t have them due to the lack of funds. It doesn’t make the work enjoyable* [Participant 8, Cape Coast].

### Processes/activities related to the delivery of integrated health services

The main issues identified under this theme were categorised into five sub-themes; health system-based challenges, national/general challenges, patient-centred care, follow ups, and THM training and research.

#### Health system-based challenges

This section explores challenges identified by participants within the processes and activities related to integrated healthcare delivery for malaria control. Two issues emerged: First, disapproval from orthodox medicine providers: Participants expressed concerns regarding negative perceptions and attitudes towards THM integration from some orthodox medicine providers. These negative views were perceived to hinder effective collaboration and patient referrals. One example cited by participants was the misconception that THM can cause kidney and lung problems. They reported efforts to address these misconceptions through clinical meetings, but with limited success.

*Another challenge we are facing, which we are trying to solve has to do with the attitudes and perceptions of the medical doctors and other orthodox practitioners. They have the perception that when you take herbal medicine, it will affect your kidneys and lung. it is a major challenge to the integration. We organise clinical meetings to explain to them, yet they are not convinced* [Participant 8, Cape Coast].

*……for the medical doctors, they don’t want to hear about THM. They think that they go to school to learn about scientific medicine. But they don’t know that currently THM providers also go through scientific training. Their opposition is one of our challenges* [Participant 3, Wa].

Secondly, participants, particularly those from the coastal and savannah regions, highlighted challenges associated with referral practices within the integrated healthcare system. They reported that orthodox medical doctors often exhibited reluctance to formally refer patients to medical herbalists. Instead, some doctors resorted to informal methods of recommending THM, blurring the lines between a formal referral and casual advice.

Participants attributed this reluctance to several factors:

- Perceived Superiority: Some medical herbalists believed that orthodox doctors held a sense of superiority, viewing them as apprentices rather than qualified healthcare providers.
- Patient Affordability Concerns: Participants suggested that doctors might hesitate due to concerns about patients’ ability to afford certified herbal medicines offered at integrated facilities, as these often require out-of-pocket payments.

*It is difficult for the orthodox medicine provider to accept and refer patients to us. For those who refer, they do it ‘backdoor’, in the form of an advice rather than as a formal referral* [Participant 6, Cape Coast].

*…the medical doctors prefer to treat malaria cases rather than referring to us…like I said, the herbal medications are cash-and-carry. So, the doctors are concerned about the cash. They are not certain that the patient will pay for the medication, and so, they prefer to treat the patient with their medication rather than referring them for herbal treatment* [Participant 8, Cape Coast].

*For my practice over ten years, no medical officer has referred a patient to me. We have a referral form that we fill. The challenge is that they frown on our referral forms. So, what we do is to refer the person verbally…. we do it in an informal setting. we don’t have a problem referring patients to the orthodox. But they will not do that. Why will they [orthodox] do that? They see themselves as superior, so why would they want to refer to us? They [orthodox providers] see us as apprentice.* [Participant 19, Wa].

#### National/general challenges

This theme highlighted issues at the national level that hindered effective management of malaria through the practice of integrated healthcare. Major national/general challenges that emerged were non-exhaustive NHIS coverage and inadequate promotional activities on THM integration. Participants narrated that the non-comprehensive nature of the NHIS (exclusion of herbal anti-malarial drugs) accounts for low patronage of services at THM clinics at integrated hospitals because clients always opt for free orthodox anti-malaria medicines rather than paying full cost for the herbal ones.

*…. the malaria medication for orthodox is on the health insurance but when you opt for the herbal medicine, then you will be paying about ghs80 or more. So, free or ghs80? The cost involved is what is accounting for the low patronage of herbal medicines for malaria in our THM clinics* [Participant 18, Kumasi].

Promotional activities play a vital role in the successful implementation of interventions. When the providers were asked to share their experiences concerning publicity of integration and its implication on malaria control, they gave an account, which suggests inadequacy of promotional activities regarding the integration programme.

*… One major challenge we are facing with this integration is the lack of publicity or awareness. Most people don’t know about this integration. The Ministry of Health should have projected the integration right from the onset. Now, when you visit these selected hospitals (integrated facilities), there are herbal practitioners available, but people do not know about that, so they do not patronise our services* [Participant 8, Cape Coast].

#### Patient-centred care

When discussing patient care, participants recounted that attending to their clients on time and spending ample time with them is something they were proud of. They emphasised that the patients really appreciated that kind of care unlike the orthodox unit, where less time is spent. The ensuing quote represent this finding:

*It is something we are very proud of at our unit. We spend a lot of time, about an hour with one patient.…. They really do appreciate that unlike the orthodox side that within 2–3 minutes they are done and don’t have time for them. So, when it comes to patient-provider relationship, it is the best in our unit* [Participant 11, Cape Coast].

#### Follow ups

We found that follow ups, which is a feature of functional health systems was well implemented by the participants. They narrated that their desire to promote the welfare of clients motivated them to follow up and conduct medical reviews to avert undesirable treatment outcomes.

*If you are interested in a case, then you follow up! There are others (clients) that I had to give my contact for them to call back. I do this to avoid any adverse effect of the treatment or medication* [Participant 2, Wa].

*… Some of the clients come for review. Through the follow ups we schedule reviews. After taking the THM, we don’t let them stay home. We schedule reviews and when they come, we test and make sure they are fine* [Participant 15, Kumasi].

*We also do a lot of follow up on our patients. We call to check up on them and find out whether they have had any side effects* [Participant 8, Cape Coast].

### THM training and research

Participants recognised that the practice of integration has boosted research on herbal remedies for treating malaria. They perceived the herbal anti-malarial medications prescribed and utilised within the integrated system to be safe and effective because they go through scientific scrutiny at the Centre for Research into Plant Medicine and/or the Nogouchi Memorial Institute for Medical Research. The quotes below reiterate this finding:

*I think it has boosted research on THM for malaria control because herbal anti-malaria drugs must be tested either at Nogouchi Memorial Institute for Medical Research or Centre for Research into Plant Medicine to verify whether it is safe for human consumption* [Participant 1, Cape Coast].

*Our THM comes from the Centre for Research into Plant Medicine; a lot of research has gone into these products, and they are safe for use. They are now safe and efficacious for treating malaria* [Participant 15, Kumasi].

In addition, participants within the forest belt reported that they usually undergo continuous professional development training. According to them, such trainings were organised by the traditional and alternative medicine directorate and involved the interpretation of scientific medical activities such as x-ray reading, understanding laboratory results and clinical emergencies. They believed that this knowledge enabled them to interpret and confirm diagnosis of diseases, especially malaria. The quotations below summarise this report:

*…. as medical herbalists we go for continuous professional development training. The training is mostly dependent on current situations. So, if there is a new herbal medication or new ways of doing things, we do that to improve upon the knowledge that we have. If there are research works going on, they update us on findings. We also do research because science is always updating. The training is mostly done by the Traditional and Alternative Medicine Directorate. They organise and get resource people to take us through it* [Participant 14, Kumasi].

*We have been going for continuous professional development (CPD) training. We do it yearly to upgrade our practice and the training largely depends on the topics that we deal with in that year. We have received training on the interpretation of x-rays, lab results and clinical emergencies…. the knowledge acquired enable us to request for lab testing and interpret the results to confirm our diagnosis for malaria* [Participant 15, Kumasi].

Interestingly, participants at the savannah belt believed that THM training focuses on exposing medical herbalists to both modern/orthodox and traditional medicines. However, the same could not be said for orthodox medicine providers where training is more focused on orthodox approaches. This unbalanced training was perceived to have created knowledge gaps among mainstream healthcare providers, leading to a lack of understanding and appreciation of the contribution of THM to healthcare delivery in Ghana.

*Some of the orthodox practitioners do not understand herbal medicine. We are trained to understand both orthodox and herbal medicines, but they are not trained that way. We have an appreciation of both sides but for them, they weren’t trained like that. They were trained one-sided and so they don’t understand herbal medicine, creating opposition from them* [Participant 2, Wa].

### Outcome/consequence of the practice of THM integration on malaria control

A key aspect of the study was to explore the perceived impact of THM integration on malaria control. Participants identified two main impacts - reduced pressure/burden on orthodox medicine providers and evidence-based THM practice leading to appropriate management of malaria. The participants perceived that their presence at public hospitals reduced pressure on orthodox medical doctors because they mostly treat malaria cases at their units and that the majority of the clients prefer herbal anti-malaria medication such as the ‘Mebeema’.

*When you take the cases we treat in our unit, malaria is among the top five. So, if the orthodox doctor was treating about 1*,*000 cases, the numbers have reduced now because most people want to use the herbal anti malaria medicine, that is, Mebeema* [Participant 11, Cape Coast].

*…. even in our facility, we have about three different drugs for malaria. The most potent one is Mebeema from Akuapem Mampong. It has been used in the system for long and the response has been very good. Lots of people who come to the unit opt for it. Clearly, our presence here at the hospital has reduced pressure on the orthodox providers* [Participant 16, Kumasi].

Besides, reducing pressure on orthodox medical doctors, participants irrespective of location, reported that the integration of THM has been beneficial because malaria cases are now appropriately treated through the evidence-based practice of their field. They believed the application of scientific clinical procedures such as correct diagnosis through laboratory testing/prescription, health education, and FDA approval of herbal anti malarial medications have led to effective malaria treatment, hence reducing prevalence. The following quotes elaborate on the views expressed by the study participants.

*I will say that about 50% of our clients come for malaria treatment. Just as they do for orthodox medicine, we also make sure that the client is taken through education, then we ask them to get tested through the lab and confirm they have malaria before we allow them to take our medication. Evidently, we apply scientific clinical procedures in treating our clients, and it has proven effective because the cases are reducing, and people are getting healed quickly* [Participant 9, Cape Coast].

*… integration has helped. …. At first, they will go to the market and get anything. But now, they (patients) come to the hospital and go through the process before receiving treatment. Malaria cases have gone down in recent times. When they come in for the medication, we also advise them to keep their environment clean and sleep under treated bed net. So, it is difficult for you to see someone come here with malaria, get treated and return to the hospital again because of malaria* [Participant 14, Kumasi].

*…. integration has helped a lot in controlling malaria in the country. I say that because the medicine has gone through FDA to be tested for its effectiveness and safety. If the patient takes the medications according to the prescription, you realise that most of them recover fully due to our medication. Many times, we are able to even treat severe malaria, so the cases are reducing* [Participant 13, Wa].

**Fig. 3:**
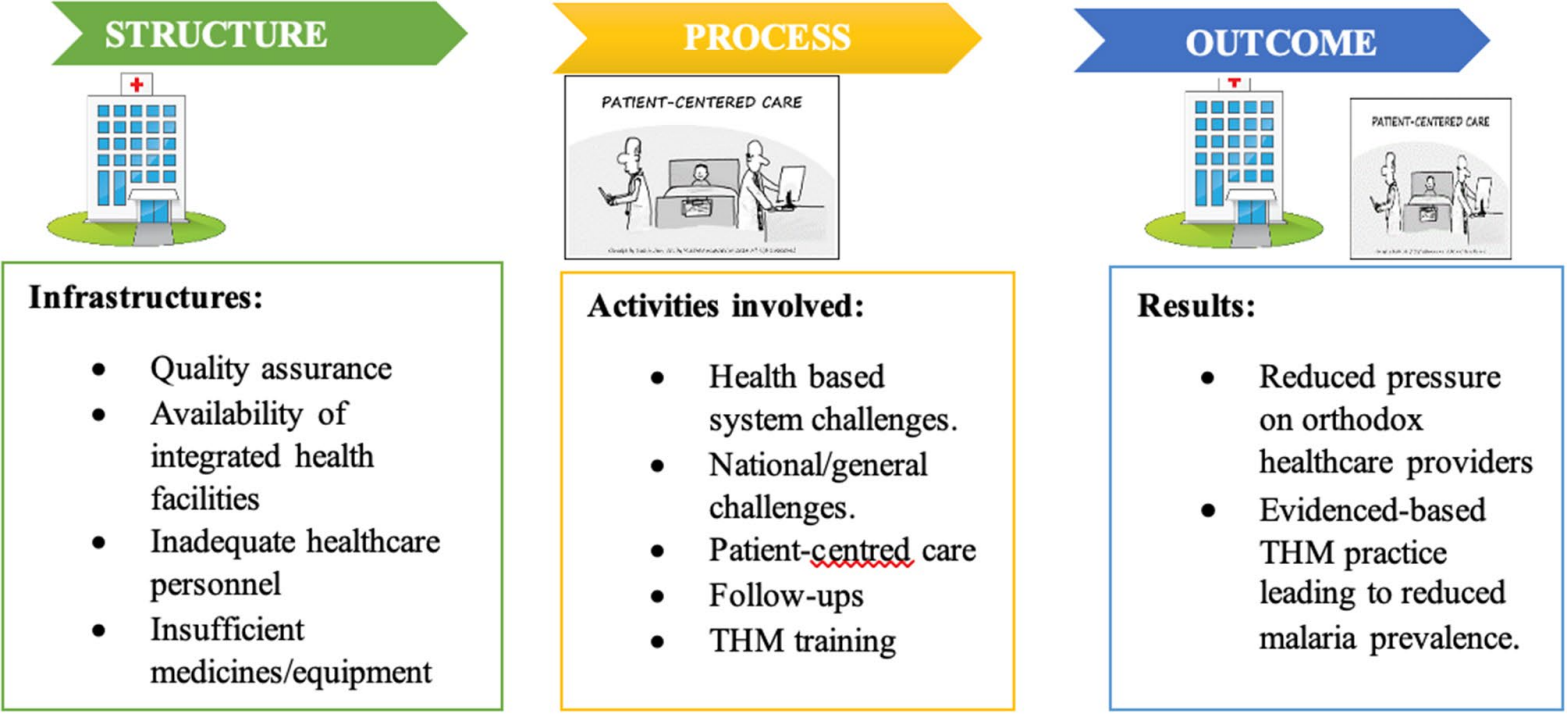
Summary of study findings guided by the framework for evaluating quality of healthcare

Figure 3 summarises the study findings guided by Donabedian framework for evaluating quality healthcare. Findings relating to infrastructure have been placed under the ‘structure’ component of the framework, while issues/activities that involved direct health service delivery have been grouped under the ‘process’ component. The effect of structure and process are presented under ‘outcome’ (Fig. 3).

## Discussion

This qualitative study employed Donabedian’s framework [12] to explore the effects of integrated healthcare on malaria control in Ghana. Thematic analysis revealed structural and process-related factors influencing the integration practice and its impact on malaria management. These factors can operate independently or interact with underlying issues.

### Structural considerations

Quality assurance, availability of integrated facilities, staffing levels, and medication/equipment availability emerged as key structural elements. The study suggests that integration promotes quality care for malaria patients. Medical herbalists, both in private clinics and integrated facilities, reported adhering to acceptable medical practices and undergoing regular FDA inspections, ensuring the quality of their services. This indicates that the traditional herbal system could be a viable alternative to the mainstream system if providers consistently follow appropriate procedures. The study findings align with those of Ampomah, Malau-Aduli [24], since the participants indicated their awareness of the practice and existence of integrated hospitals in Ghana. However, in the current study, the participants felt that the number of integrated hospitals was insufficient compared to the national population. They attributed this inadequacy to low governmental support for integration and low community utilisation of these services. With a growing population and a stagnant number of integrated facilities, access for many Ghanaians, particularly in rural areas, would be limited. The results further showed gender discrepancy among medical herbalists in Ghana, where there are more males than female providers; however, this might not serve as a hindrance to access (not prevent patients especially females from seeking care at integrated hospitals) because malaria is not an intimate health problem.

### Challenges with personnel, equipment, and procurement

Findings revealed that medical herbalists handle a wide range of administrative and dispensary tasks to maintain service delivery within the integrated system. This is likely due to insufficient number of registered medical herbalists and limited support from orthodox healthcare providers, who may lack expertise in THM. This highlights a critical gap in personnel recruitment and deployment for successful integration implementation. These findings resonate with previous studies emphasising the importance of training/professional development for both providers to aid quality service delivery [25, 26].

Another key issue was inadequate equipment and medication supply within THM units of integrated facilities. While participants reported access to some laboratory services, those in the coastal region lacked essential computers and software for e-health practices. Donabedian’s framework emphasises the importance of equipment/drug supply and its availability for quality healthcare [12]. Participants in the forest zone reported disruptions due to insufficient herbal anti-malarial medications, attributing it to bureaucratic procurement processes. The issue of shortage of certified herbal medications aligns with findings from an earlier study [24] and highlights a problem prevalent in low- and middle-income countries [27]. A re-evaluation of procurement procedures within the healthcare system is necessary to eliminate bureaucratic obstacles hindering appropriate treatment delivery.

#### Deliberations on process

Participants identified challenges related to healthcare system processes. A prominent concern was the disapproval of orthodox healthcare providers towards THM due to negative perceptions. This has also been reported in previous studies [11, 24, 28, 29] and hinders collaboration in managing malaria cases. Addressing these “erroneous notions” requires increased education for both healthcare provider groups. Another challenge was the prevalence of informal referrals instead of formal ones, echoing findings from Ashanti [11, 30], Brong Ahafo [28], and Northern parts of Ghana [31]. This potentially leads patients to seek care from uncertified THM providers, jeopardising their health. To improve access to safe herbal anti-malarial treatment and enhance user well-being, it is crucial to revise or develop integrated policies to revamp referral structures.

The limited scope of the NHIS and inadequate promotion of integrated healthcare were perceived by participants in this study as national challenges. The NHIS, designed to address healthcare access issues [32], was found to be insufficient for covering herbal anti-malarial therapies within integrated facilities. This aligns with previous studies among health service users [29], providers, and hospital administrators [30], where NHIS limitations hindered patients’ access to THM clinics, consequently undermining the program’s effectiveness. Policy reform towards a more patient-centred healthcare system is needed to ensure equitable access.

The Donabedian framework emphasises patient-provider respect and trust [12]. This was evident in our study, as participants described their patient-centred approach, including extended consultations and follow-up care to promote overall well-being and minimise adverse treatment effects. This finding resonates with research in Ghana’s Ashanti region where users [29] and providers [24] reported a compassionate approach to healthcare delivery. Such an attitude might encourage users to continue seeking care from THM providers. The study confirmed that medical herbalists across facilities receive formal training. Participants in the forest zone attributed their ability to interpret lab results and diagnose effectively to ongoing professional development programs. However, those in the savannah belt felt hampered in collaborating with orthodox counterparts due to imbalanced training. Medical herbalists receive training on both traditional and modern healthcare, while their orthodox counterparts lack exposure to THM. This perceived knowledge gap hinders appreciation for traditional therapies and their role in healthcare delivery. Studies [24, 28, 30], have shown similar findings, where orthodox practitioners acknowledge their lack of THM knowledge as a barrier to collaboration. While trained medical herbalists can expand Ghana’s healthcare workforce, unbalanced training may disrupt service delivery, especially for malaria patients [30].

#### Outcome – effects of structure and process on malaria control in Ghana

The Donabedian framework suggests that good structures and processes should lead to positive health outcomes [12]. While some structural and process factors were not ideal, most participants believed that integrated healthcare has reduced pressure on orthodox providers, who frequently manage malaria cases. The availability of medical herbalists in public hospitals was seen to contribute to decreased malaria prevalence in Ghana through evidence based THM practices. This finding offers a novel contribution to literature.

#### Practice implications

The integration of herbal therapies into mainstream healthcare reflects an attempt to regulate and manage healthcare users, providers, and herbal medication use. Patients are directed towards trained medical herbalists who follow established medical standards, including scientific manufacturing, packaging, and administration under medical facility supervision. This aligns with a Vietnamese study [33] which described herbal medicine modernisation as a means to safeguard public health. While integration primarily aims to protect public health and well-being, it also serves to regulate herbal remedy usage within a population. As this research demonstrates, the Ghanaian government aims to provide patients with a single point of access for malaria treatment through integration. However, achieving this health goal is hindered by national and health system-based challenges.

#### Strengths and limitations

This study is one of the few to explore the contribution of integrated healthcare to malaria control in Ghana. A key strength lies in the inclusion of medical herbalists from diverse regions (coastal, forest, and savannah), providing insights from a crucial stakeholder group directly involved in healthcare delivery. The study offers valuable knowledge by examining the contribution of integrated medicine to malaria control. However, limitations exist. The small sample size, chosen through non-probabilistic sampling, reduces the findings’ representativeness and generalisability. While the study successfully recruited medical herbalists as appropriate participants, it is important to acknowledge the potential influence of personal biases on their perspectives. This could have led to overestimation or exaggeration of certain narratives. Furthermore, the exclusive use of face-to-face individual in-depth interviews as the data collection method may have limited the generalisability and reliability of the study findings.

## Conclusions

Key findings indicate high awareness of integration among Ghanaian medical herbalists. Participants believe that quality assurance, designated facilities, patient-centred care, follow-up practices, and continuous professional training for medical herbalists contribute to reduced burden on orthodox healthcare providers and evidence-based THM practices leading to improved malaria management. However, structural barriers (inadequate personnel, medication, and equipment) and process challenges (limited NHIS coverage, inadequate promotion, ineffective referrals) hinder integration’s contribution to malaria control. These findings, therefore, offer the model or baseline information and a platform for further discussion towards improving the Ghanaian integrated health system to serve as a tool for achieving a malaria free country.

Eradicating malaria through integrated healthcare requires policy modifications and improved implementation. Hence, we offer the following recommendations: the creation of herbal department at KNUST is impressive, however, the educational policies need to be revised to expand herbal medicine training to other institutions and incorporate herbal medicine into the curriculum of biomedical healthcare providers. This might enhance the conventional healthcare practitioners’ understanding on herbal medicine and improve collaborations between medical herbalists and their biomedical colleagues. The government needs to formulate policies that would increase public awareness about the practice of integrated healthcare (availability of medical herbalists and certified herbal anti-malarial medications in government hospitals) among health service users. Increased awareness could lead to improved access to such services. Future studies could focus on developing healthcare frameworks tailored to eliminate infectious diseases, particularly malaria, that prioritise quality service delivery within an integrated system.

## Electronic Supplementary Material

Below is the link to the electronic supplementary material.

Supplementary Material 1

Supplementary Material 2

## Data Availability

All data generated or analysed during this study are included in this published article [and its supplementary information files].

## Abbreviations

COREQ: Consolidated Criteria for Reporting Qualitative studies
CSRPM: Centre for Scientific Research into Plant Medicine
FDA: Food and Drug Authority
HIV: Human Immunodeficiency Syndrome
KNUST: Kwame Nkrumah University of Science and Technology
NHIS: National Health Insurance Scheme
THM: Traditional Herbal Medicine
UCC: University of Cape Coast
UDS: University of Development Studies
WHO: World Health Organization
