# Supplementary material for: Exploring the contribution of integrated healthcare practices to malaria control in Ghana: perspectives of medical herbalists
Source: BMC Complement Med Ther. 2025 Jan 14;25:11. doi: 10.1186/s12906-025-04746-9 (PMC11734445; doi:10.1186/s12906-025-04746-9)
Supplement: Supplementary file 1 — Supplementary Material 1 [file 12906_2025_4746_MOESM1_ESM.docx]

Interview Questions: Medical herbalists.

**Background characteristics of participants:**

Name of Metropolis/Municipality.....................................................................................

Age.....................................................................................................................................

Sex......................................................................................................................................

Marital status…………………………………………………………………………….

Tribe/ethnic origin.............................................................................................................

Educational level................................................................................................................

Profession………………………………………………………………………………...

Specialty of participant…………………………………………………………………..

1. Please, what role do traditional herbal medicine play in healthcare delivery in Ghana?
2. Are you aware of the practice of TM integration in Ghana?
3. As a health practitioner, can you share with me the contribution of the practice of integrated healthcare in promoting effective malaria control in Ghana?
4. Please, are there any challenges associated with the practice of integration and the control of malaria in Ghana?
5. In your opinion, has the practice of integrated healthcare helped in controlling malaria in Ghana?
6. Please, in what ways do you think Ghana as a country could achieve effective malaria control through the practice of TM integration?
7. Do you have additional information regarding the topic that you would like to share with me?

**Thank you for your time and contributions.**
